# A phylogenetic review of cancer resistance highlights evolutionary solutions to Peto’s Paradox

**DOI:** 10.1590/1678-4685-GMB-2022-0133

**Published:** 2022-12-05

**Authors:** Mariana F. Nery, Mathias Rennó, Agnello Picorelli, Elisa Ramos

**Affiliations:** 1 Universidade de Campinas, Departamento de Genética, Evolução, Microbiologia e Imunologia, Campinas, SP, Brazil.

**Keywords:** Cancer, evolution, Peto’s paradox, mammals

## Abstract

Cancer is a genetic disease present in all complex multicellular lineages. Finding ways to eliminate it is a goal of a large part of the scientific community and nature itself. Early, scientists realized that the cancer incidence at the species level was not related to the number of cells or lifespan, a phenomenon called Peto's Paradox. The interest in resolving this paradox triggered a growing interest in investigating the natural strategies for cancer suppression hidden in the animal's genomes. Here, we gathered information on the main mechanisms that confer resistance to cancer, currently described for lineages that have representatives with extended longevity and large body sizes. Some mechanisms to reduce or evade cancer are common and shared between lineages, while others are species-specific. The diversity of paths that evolution followed to face the cancer challenge involving coding, regulatory, and structural aspects of genomes is astonishing and much yet lacks discovery. Multidisciplinary studies involving oncology, ecology, and evolutionary biology and focusing on nonmodel species can greatly expand the frontiers of knowledge about cancer resistance in animals and may guide new promising treatments and prevention that might apply to humans.

## Introduction

Cancer is a genetic disease that occurs during unregulated cell division and may form malignant tumors. These malignant tumors are caused by mutations induced by environmental factors or inherited. A study has suggested that errors during DNA replication play a predominant role in cancer development (Tomasetti *et al.*, 2017). As replication is a crucial process in multicellular organisms, several tumor suppressor mechanisms evolved to prevent the spread of somatic mutations in a cell population, such as cell cycle checkpoints and apoptosis. However, despite these defense mechanisms, cancer still occurs at high rates in some species, particularly humans (Albuquerque *et al.,* 2018).

Cancer has been observed in all seven times that complex multicellularity emerged (Aktipis *et al.*, 2015), suggesting that since the origins of those branches, cancer-like behavior imposed a selective pressure that guided the evolution of multicellular organisms. Essentially, complex functional multicellularity requires the suppression of cell-level fitness to promote organism-level fitness because, looking through the cell perspective, the best strategy to maximize their fitness in the short term would be cheating the intercellular cooperative rules (Aktipis *et al.*, 2015).

A study of known cancer-related genes showed that cancer-suppression adaptations, especially for gatekeeper genes, have arisen rapidly in the earliest metazoan's common ancestors, closely matching the origin of multicellularity (Domazet-Lošo and Tautz, 2010). The matching implies that these genes are part of the core traits that enabled the emergence of multicellular organisms and highlighted the importance of the evolution of controlling the individual cell's selfish behavior. In addition, given the multiple roads that have led to different origins of multicellularity, intercellular cooperation and cheating suppression must have evolved many times across the Tree of Life.

While most, if not all, vertebrate species are affected by some form of cancer, some are at more risk than others (Effron *et al.*, 1977; Abegglen *et al.*, 2015). The Doll-Armitage multistage theory (Armitage and Doll, 1954) predicts carcinogenesis as a multistage process of accumulation of genetic and epigenetic mutations in a mitotic cell, which leads to the wide acceptance that cancer prevalence should be a function of the number of cell divisions per time. In other words, cancer risk should rise due to more cell divisions and more prolonged exposure to endogenous and exogenous stressors. Thus, larger and long-lived animals should be more susceptible to developing cancer. However, it has been verified that interspecies cancer rates do not correlate with body mass or lifespan. The mismatch between theoretical prediction and observation is known as Peto's Paradox (Peto *et al.*, 1975; Nunney, 1999). This paradox can be best understood when we consider that evolution has compensated for the increase in the risk of cancer development with the selection of more and better mechanisms for cancer suppression.

In this context, the comparative and multidisciplinary approaches may provide exciting insights into shared and specific methods of cancer evasion, as many mechanisms have evolved to decrease cancer risk across the Tree of Life. Accordingly, this review aims to briefly discuss some evolutionary theories behind cancer and unravel most anticancer mechanisms reported thus far, given the growing data on the evolution of cancer suppression in different lineages.

## The Evolutionary Theory Behind Cancer

It is often postulated that natural selection has a fundamental role in evolving efficient anticancer defenses giving rise to many different mechanisms in independent lineages to keep multicellular life viable. However, natural selection has some limitations in cancer suppression - as we can see that no organism demonstrates complete flawless anticancer adaptations. Some reasons have been proposed to explain why organisms remain vulnerable to cancer over evolutionary time.

One first reason is that selection is slow and constrained by millions of years of the phylogenetic history of a lineage. Evolution by natural selection takes time to fixate new alleles in a population. Consequently, organisms are adapted to past, not present, circumstances. It is relevant when organisms face a mismatch of scenarios and environments where extensive and unpredictable changes drastically alter the fitness landscape. An excellent example of this phenomenon is that subpopulations of light-skinned individuals who migrated to equatorial zones have disproportionately higher rates of skin cancer due to sun exposure because, in their habitat of origin, they had different evolutionary pressures (Jablonski and Chaplin, 2010). Another example is when the situation involves an oncogenic pathogen organism, the coevolution wages a host-pathogen arms race creating cycles of escalating responses and counter-responses. This keeps host defenses in suboptimal states because they are costly, and pathogens evolve much faster than larger organisms (Arthur and Jobin, 2011; Ewald and Ewald, 2013).

Another reason is that "evolution cannot make Darwinian Demons". A Darwinian Demon is a hypothetical organism that would simultaneously maximize all aspects of fitness and exist if there were no limitations to what evolution can produce. However, evolution has many constraints and trade-offs. For example, the evolution of a trait toward its maximum theoretical fitness will eventually hit a drift barrier because the closer a trait comes to hypothetical perfection, the smaller the fitness advantages (Lynch, 2010). It helps explain the incidence of rare pre-reproductive familial cancers such as retinoblastomas and neuroblastomas caused by inherited vulnerable alleles that could not be selected against fast enough to cause these diseases to become clinically irrelevant (Nunney and Muir, 2015).

Finally, trade-offs commonly come as a cost in the fitness contribution of another trait. Some can be nullified or minimized under certain conditions, while others are unavoidable. This can be illustrated by the trade-offs between cancer and aging provoked by the tumor suppressor gene (TSG) p53. Extra copies of p53 protect against cancer in genetically modified mice because they exhibit apoptosis mechanisms more sensitive to DNA damage. However, if the additional copies are constitutively expressed, this protection has the cost of an accelerated rate of aging (Tyner *et al.*, 2002). However, this trade-off is nullified if those p53 extra copies are placed under proper regulatory control by its endogenous promoters (García-Cao *et al.*, 2002).

There is also a trade-off in how well the immune system can detect and discriminate normal from cancer cells because they are closely related and, thus, very similar (Mapara and Sykes, 2004). While activation of immune surveillance and inflammation is crucial for dealing with potentially oncogenic infections or cheater cells, exacerbating it can lead to autoimmune diseases and tissue damage and make them more vulnerable to cancer. Paradoxically, as it might seem, the opposite can also lead to cancer (Coussens and Werb, 2002; De Visser *et al.*, 2006).

These examples show that the emergence of complex multicellularity has brought a difficult challenge in which the solution is subject to the limitations of the evolutionary process. However, although the war against cancer is far from being won in an evolutionary context, many victories have already been described due to the emergence and maintenance of new genetic and cellular strategies to combat uncontrolled cell division in several species.

Life history matters

Ecology and life histories have a significant role in the evolutionary outcome of a given lineage. For example, old-age animals are rare in the wild because natural mortality occurs due to extrinsic factors such as infection, predation, competition, or starvation. This means that natural selection will have limited opportunity to directly influence the senescence process and the diseases that will come with age, such as cancer (Kirkwood and Austad, 2000). In other words, the selection force is weak with increasing age in circumstances of high extrinsic mortality rates. As formulated by Medawar's "Mutation Accumulation Theory", this will result in a 'selection shadow' that allows alleles with late deleterious effects to accumulate over generations.

Moreover, pleiotropic genes with harsh effects at later ages would be favored by selection even if they have only minor benefits early in life, known as the "Antagonistic Pleiotropy Theory" (Williams and Nesse, 1991). This happens because the contribution to fitness is a composite of the effect's size and the probability of surviving to be affected by it. Early in life, a small beneficial effect can outweigh a deleterious late impact, even if this results in senescence and death (Kirkwood and Austad, 2000; Roper *et al.*, 2021). DeGregori (2011, 2017) expands this view, arguing that it is also essential to account for the tissue microenvironmental changes - dependent on age - in altering selective pressures that ultimately dictate cancer incidence.

According to life-history theory, animals reach local fitness peaks through different modes and rates of reproduction, growth, maintenance, and survival. These traits are subjected to trade-offs because of finite resources, influencing what strategies will be selected according to ecological and phylogenetic variables that dictate which phenotypic configuration would be optimal for that group (Stearns, 1989).

Using this theoretical framework, we can predict that adaptations such as the capacity to fly, protective shells, larger body sizes, and subterranean behavior, for example, may reduce the levels of extrinsic mortality, thus raising the selective pressure for genes related to somatic maintenance, which is likely to result in extended longevity and cancer resistance. In addition, an evolutionary model based on life-history theory can help us make more assertive testable hypotheses, as shown by Brown *et al.* (2015).

In this context, an increasing body of research has found that some life-history traits can help to explain the differences in cancer prevalence within different species. For example, Vincze *et al.* (2022) found a positive relationship between a carnivorous diet and cancer risk. In addition, Boddy *et al.* (2020) found a positive relationship between litter size and cancer prevalence in mammals, while Møller *et al.* (2017), studying the prevalence of cancer in birds, demonstrated a significant negative relationship with better immunity and slower developmental rates controlled for body size. These results reinforce the importance of considering life histories when studying cancer prevalence.

Cancer in a phylogenetic context

Cancer and cancer-like phenomena are extraordinarily ubiquitous across the tree of life. All vertebrates develop cancer, and the fossil record suggests they always have cancer (Rothschild *et al.*, 1999). The oldest fossil record of vertebrate cancer is approximately 240 million years old (Haridy *et al.*, 2019). However, cancer incidence among vertebrate species is not equal; for example, it is lower in birds and reptiles than in mammals (Effron *et al.*, 1977; Kitsoulis *et al.*, 2020). Furthermore, as mentioned before, this incidence does not scale with body size or lifespan. The absence of this relationship has attracted the scientific community's attention, and cancer in wildlife has been receiving more attention over the last few years.

Cancer is well monitored in human populations and domestic animals (Meuten, 2002). However, despite its value, data on the impact and prevalence of cancer in wildlife is still largely unknown and likely to be underreported and understudied for several reasons: long-term mortality investigations in wildlife are rare, and it is challenging to detect cancer in affected living individuals, and if cancer results in mortality, carcass decomposition may limit discovery (Pesavento *et al.*, 2018; Baines *et al.*, 2021). Recent efforts have been made to report cancer prevalence in other species besides human. Madsen *et al.* (2017) performed a comprehensive and updated review on the prevalence and etiology of cancer in wild and captive animals. Later, Albuquerque *et al.* (2018) reviewed cancer incidence and types from various species. More recently, Vincze *et al.* (2022) characterized cancer incidence across a broad taxonomic range in mammals using the most extensive dataset. Their results provide unequivocal support for the validity of Peto's paradox in mammals*,* as these species differ broadly in their cancer rates (Vincze *et al.*, 2022). Still, available data is anecdotal, and we lack an accurate recording of cancer incidence in other non-mammalian taxonomic groups and most wildlife populations that are not closely monitored. Cancer statistics along the tree of Life is an essential source of information to further explore Peto's Paradox through the lens of evolutionary and comparative biology, as evolution has come to multiple solutions to delay and suppress cancer independently and under different circumstances.

In recent years, efforts have been devoted to solving Peto's paradox. Some hypotheses could explain how organisms could overcome cancer despite a more significant number of cell divisions over a lifetime, but most of these solutions are only theoretical (Roche *et al.*, 2012; Maciak and Michalak, 2015). Despite being largely accepted as an intriguing paradox that deserves attention to better understand cancer resistance, some authors claim that the oversimplistic hypotheses behind Peto's paradox are inaccurate. Ducasse *et al.* (2015) argue that Peto's paradoxical legitimacy should include ecological, environmental, and behavioral factors (Ducasse *et al.*, 2015). Additionally, these authors emphasize the importance of organ-level comparisons when investigating the variation in cancer resistance since previous studies have shown that differences in cancer risk may be explained by the number of stem cells in the tissue (Tomasetti and Vogelstein, 2015) and by the complexity of cancer signaling networks (Breitkreutz *et al.*, 2012).

While the validity of Peto's Paradox is still in debate, investigations on the evolution of cancer defenses are of fundamental importance. Moreover, this evolutionary conundrum has fostered much progress in our understanding of cancer resistance and fueled the research on the evolution of anticancer mechanisms, generating important advances in our knowledge of cancer in other nonhuman animals. As a result, many exciting results have been reported in the last few years, and the potential for comparative oncology studies is very promising.

## Mechanisms to Evade Cancer

**Figure 1 -:**
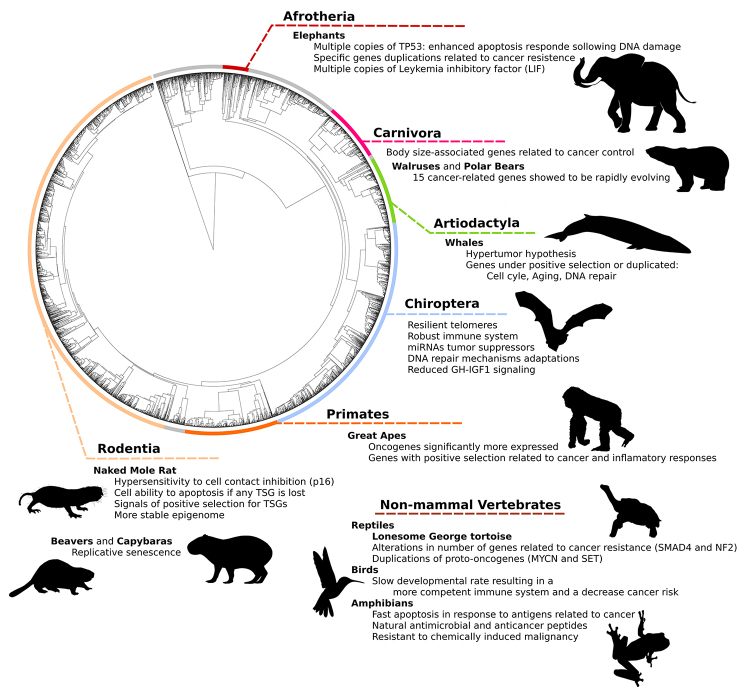
Summary of the main genetic mechanisms related to cancer resistance in vertebrate lineages.

Interest in investigating the molecular mechanisms of natural cancer resistance in nonmodel species has been growing considerably in recent years. Scientists realized that knowledge of these mechanisms might be a powerful weapon in developing human cancer-preventive and therapeutic strategies. Here, we will discuss the progress achieved in identifying mechanisms of cancer resistance in different lineages that show extended longevity and large bodies compared to their phylogenetic counterparts (Figure 1).

Elephants

Currently, only three species of elephant exist: the African bush elephant (*Loxodonta africana*), the African forest elephant (*Loxodonta cyclotis*), and the Asian elephant (*Elephas maximus*). As the largest extant land mammals living on Earth, elephants have recently caught the attention of the scientific community in an attempt to understand Peto's Paradox (Abegglen *et al.*, 2015; Caulin *et al.*, 2015; Sulak *et al.*, 2016; Vazquez *et al.*, 2018; Tollis *et al.*, 2021; Vazquez and Lynch, 2021). The first indications that elephant lineage contains genetic strategies to enhance cancer protection mechanisms came from studies by Abegglen *et al.* (2015) and Sulak *et al.* (2016). Abegglen *et al.* (2015) found that elephants have a lower cancer rate than expected based on their body size compared with other mammalian species. This was related to multiple copies of the TP53 gene, widely known as a crucial tumor suppressor gene (TSG), preventing the growth and survival of potentially malignant cells (Ryan *et al.*, 2001). While most mammals have only one TP53 copy in their genome, the African bush elephant genome contains 19 extra copies of TP53, 9 to 20 copies were identified in the Asian elephant genome, and 21 to 24 copies were found in the African forest elephant genomes (Abegglen *et al.*, 2015; Caulin *et al.*, 2015; Sulak *et al.*, 2016; Tollis *et al.*, 2021). Moreover, the extinct woolly mammoths and the straight-tusked elephant genomes also presented between 19-28 and 22-25 TP53 copies, respectively (Tollis *et al.*, 2021).

Interestingly, the extra 19 copies of TP53 genes for the African bush elephant lack true introns, indicating that those genes likely originated after a process of retrotransposition (Abegglen *et al.*, 2015; Gaughran *et al.*, 2016; Muir, 2016; Sulak *et al.*, 2016), and those extra copies started being called TP53 retrogenes (TP53 RTGs). Later, Sulak *et al.* (2016) confirmed that some TP53 retrogenes are transcribed and responsible for an enhanced apoptosis response following DNA damage. Additionally, it was proposed that their protein products, even lacking part of their original structure, could act on the stabilization of the original p53 (Abegglen *et al.*, 2015; Seluanov *et al.*, 2018). We also cannot rule out the possibility of TP53 copies having some novel - and still unknown - function.

These studies imply that the additional copies could be related to the increased size of elephants. However, recent research found evidence that challenges this idea. Vazquez and Lynch (2021) found that duplication of TSGs was common in all Afrotherians, even those with small body sizes. This suggests that duplication of TSGs may have preceded the evolution of species with large body sizes. Moreover, very recently, Nunney (2022) evaluated TP53 RTGs and argued that the additional tumor suppressor gene activity could improve longevity in this species - as suggested by the multistage evolutionary model - the extra copies of TP53 probably accumulated after a process of duplication followed by random genetic drift. Hence, the resistance to cancer may not be related to an increased number of TP53 RTGs but to the maintenance of at least one of these copies with increased expression (e.g., RTG 12) together with enriched duplications in other pathways related to cancer resistance observed in proboscideans (Vazquez and Lynch, 2021; Nunney, 2022).

In addition to TP53, another protein has been studied to explain the success of the Elephantidae family in dealing with cancer: the multifunctional interleukin-6 class cytokine leukemia inhibitory factor (LIF). This protein is known to have functions related to tumor suppressors or acting as an oncogene. Like TP53, numerous duplicate LIF pseudogenes were found in the genomes of elephants and the genomes of their closest related lineages: hyrax and manatee. This group presents seven to 11 additional copies of LIF. From these copies, at least one LIF6 is expressed in elephant cells. The overexpression of LIF6 induces apoptosis and is required for the enhanced response to DNA damage in elephants. LIF6 is considered a "zombie" LIF gene due to the reanimation of its function after a pseudogenization process and because it is a cell killer (Vazquez *et al.*, 2018). Moreover, the LIF6 functionalization in the stem lineage of proboscideans coincides with the rise of large sizes, advocating for an important contribution of this gene to the evolution of cancer resistance in this lineage (Vazquez *et al.*, 2018).

A genomic scan (Vazquez and Lynch, 2021) also indicates other gene duplication events related to cancer resistance in Proboscideans. For example, they identified specific duplications of the COX20, LAMTOR5, PRDX1, STK11, BRD7, MAD2L1, BUB3, UBE2D1, SOD1, MAPRE1, CNOT11, CASP9, CD14, and HMGB2 genes (Vazquez and Lynch, 2021), most of which are involved in apoptosis, cell cycle regulation, DNA damage repair, cell resistance to oxidative stress and TP53 regulation.

These studies presented significant contributions and highlighted the role of the Elephantidae lineage in the study of Peto's Paradox. However, some limitations still need to be addressed. Including high-quality genomes for as many species as possible within the Afrotheria group is necessary to avoid bias in conclusions, as occurred in studies that compared only a few species. In addition, the lower estimation of the number of copies for the Asian elephant and the extinct representatives of Elephantidae could be an artifact of the lower quality of their genomes, which could be solved with the use of long sequencing to improve assembly (Tollis *et al*., 2021).

Moreover, understanding cancer resistance in elephants could also be beneficial by studying the molecular basis of the slower metabolism in these species because a slow metabolism is known to be related to lower mutations and cellular damage (Seluanov *et al.,* 2018). On this matter, Dang (2015) reviews the fundamental aspects of cancer cell metabolism and provides evidence that metabolic rates vary inversely with body mass in mammals. Besides that, the author supports the hypothesis that metabolism drives tumorigenesis and claims that a metabolic basis should be considered for Peto’s paradox (Dang, 2015). However, the relationships and discussions are still only theoretical, and empirical data on how metabolism directly contributes to cancer incidence is lacking. Finally, comprehensive gene expression data for the pathways found above are necessary to ascertain that duplicate genes are expressed and thus play an active role in cancer resistance in elephants (Vazquez and Lynch, 2021).

Whales

Whales are known for having reached the largest body mass in the history of life and displaying some of the longest-living species of mammals. Aligned with the observations of a few cetaceans with cancer, which supports Peto's Paradox, it is expected that evolution in this group has shaped anticancer mechanisms. However, we are only beginning to uncover some potential molecular bases of this resistance.

Many genomes of giant whales have been sequenced thus far but did not reveal duplications of TP53 similar to those in elephants, suggesting that they evolved different anticancer adaptations. Comparative genomics in the bowhead whale (*Balaena mysticetus*), the longest-lived whale, identified genes under positive selection and specific mutations in genes linked to cancer, aging, the cell cycle, and DNA repair, but without conclusive experiments (Keane *et al.*, 2015). Likewise, genome analysis of the humpback whale (*Megaptera novaeangliae*) genome found a strong selection of pathways linked to cancer, such as the cell cycle, cell signaling and proliferation, and duplications in genomic portions containing genes related to apoptosis (Tollis *et al.*, 2019).

Tejada-Martinez *et al.* (2021) reported signals of positive selection in seven TSGs: CXCR2, ADAMTS8, ANXA1, DAB2, DSC3, EPHA2, and TMPRSS11A. Moreover, they revealed that the turnover rate of TSGs was almost 2.4 times faster in cetaceans than in other mammals, showing 71 duplicated genes in at least one of the Cetaceans species. Most duplication events and positively selected genes were identified in the lineage of mysticetes, the large baleen whales, suggesting that they have evolved additional anticancer mechanisms. Remarkably, further functional analysis highlighted that those genes found to be duplicated or altered by positive selection are commonly associated with different types of neoplastic diseases and the regulation of senescence, cell proliferation, and metabolism (Tejada-Martinez *et al.*, 2021).

Nagy *et al.* (2007) raised an interesting hypothesis to resolve Peto's paradox in whales: they predicted that cancer might be more common and less lethal in large organisms simply because malignant tumors would need more time to reach lethal size. The main idea is that malignant tumors may be parasitized by a more aggressive competitor kind of malignant cell lineage. In this competition, the more aggressive cells are incapable of secreting enough tumor angiogenic factors (TAFs). Thus, this cell lineage takes advantage of the vascular infrastructure built by the TAF competent cells leading to a depletion of oxygen and nutrient levels within the tumor, affecting the tumor's growth. This dynamic may keep cancer at a sublethal size or even damage it until a point of inviability. In this scenario, baleen whales could present the expected cancer rate for larger organisms, and the hypertumor hypothesis would explain the negative correlation between body size and cancer risk. The authors tested this hypothesis using mathematical models and computer simulations (Nagy *et al.*, 2007). The simulations showed that hypertumors kept tumors to sublethal size and that tumors take more time in larger organisms to reach lethal sizes. In other words, the mortality rate negatively correlates with body mass. However, to our knowledge, these predictions based on computer simulations have not been empirically investigated.

Our current knowledge on the molecular basis of cancer resistance in whales points to solutions involving the duplications of tumor suppressor genes and accelerated evolution in cancer-related genes and aging, which show signs of positive selection. Nevertheless, no empirical data are available for whales. Then, we still do not understand how the positively selected genes or the duplicates acted as a cancer defense mechanism. Nevertheless, the whale's genomes promise many exciting discoveries and endure the idea that large and long-living species have evolved different and independent mechanisms to suppress cancer.

Great apes

As in other mammalian lineages, the maximum life span and body mass are correlated in primates, and the great apes are the largest body size and long-lived species among them. Tejada-Martinez *et al.* (2022) investigated the molecular evolution in coding genes and cis-regulatory sequences and gene expression evolution related to the development and maintenance of maximum lifespan and body size in the great apes and their association with pathways related to cancer suppression. They found only five genes with positive selection signals for the great ape lineage (IRF3, SCRN3, DIAPH2, GASK1B, and SELENO), all of which have functions related to cancer development and inflammatory responses. Additionally, a set of oncogenes was significantly more highly expressed in apes than in other primate species. Of these oncogenes, 22% present an ape-specific enhancer in their surroundings. The authors also identified footprints of evolution related to SINE-Vntr-Alu (SVA) insertions and LTR transposons, reinforcing the importance of the action of these transposable elements in the evolution of great apes' gene-regulatory networks, especially in humans (Trizzino *et al.*, 2017). The results of Tejada-Martinez *et al.* (2022) show that the evolution of strategies for cancer resistance in the primate lineage is quite diverse, with modifications that can be found at the coding, expression, and regulatory levels, and that although the great apes lineage provides evidence of specific changes capable of giving greater longevity to the species of the group, the understanding of the relationship with cancer resistance is still developing for nonhuman species and needs to be further investigated.

Rodents

The naked mole-rat (*Heterocephalus glaber*) is the longest-living rodent, reaching up to 30 years, and is virtually cancer-free. These intriguing subterranean rodents, the only eusocial mammal known, have been extensively studied because of their high resistance to cancer, and multiple mechanisms have been described.

Naked mole rats have hypersensitivity to cell contact inhibition. Due to early activation of the p16 pathway, cells stop dividing at much lower densities than the mouse and human cells, reducing cancer risk (Seluanov *et al.*, 2009). Additionally, naked mole rats have a more stable epigenome, which can resist reprogramming associated with malignant transformation (Toole, 2004). Another anticancer mechanism in naked mole rats is that their cells perform apoptosis when they sense the loss of a single tumor suppressor, such as p53, RB1, or p19. On the other hand, this inactivation in human and mouse cells leads to cell proliferation (Rangarajan *et al.*, 2004; Seluanov *et al.*, 2009). Furthermore, Cdkn2a-Cdkn2b, which is a rapidly evolving locus that contains critical tumor suppressor genes, has signals of positive selection (Kim *et al.*, 2011) and a unique structure in naked mole-rats, providing extra cancer protection through specific products generated by alternative splicing (Tian *et al.*, 2015). All these layers of tumor-suppressive adaptations contribute to cancer resistance in this remarkable rodent.

There is a positive correlation between telomere length and telomerase (TERT) activity in mammals. TERT plays a key role in carcinogenesis by maintaining telomere length and allowing cells to prevent senescence (for a review, see Dratwa *et al.,* 2020). Evidence suggests that replicative senescence induced by telomere shortening is a tumor suppressor mechanism (Ségal-Bendirdjian and Geli, 2019), even though this role is still debated (see Chan and Narita, 2019; Calcinotto *et al.*, 2019). TERT inactivation occurred early in placental mammals, but it was reactivated in the stem rodent lineage and is active in the most recent small rodent species (Gomes *et al.*, 2011). However, it was later inactivated in some lineages with large body masses, such as beavers (*Castor canadensis*) and capybaras (*Hydrochoerus hydrochaeris*). Vedelek *et al.* (2020) found that TERT inactivation in large-bodied rodents such as beavers occurs without a GAPBA transcription factor, which plays a key role in TERT. The authors argue that TERT inactivation in these large rodent species strengthens the hypothesis of replicative senescence as a tumor suppressor mechanism (Vedelek *et al.*, 2020). The compelling evidence that inactivation in TERT promoter has the potential to be an additional mechanism to solve Peto’s paradox should foster scientific attention to address the question of whether senescence may be a resistance mechanism varying among species.

Bats

Bats seem to master many of the environmental challenges: they are the only truly flying mammals, echolocate, have a robust immune system, and have exceptional longevity given their body size (Teeling *et al.*, 2018). Therefore, the success of this interesting group from an evolutionary point of view in presenting mechanisms that contribute to cancer resistance would not be a surprise. However, although bats constitute a pivotal group from an evolutionary perspective, there is still little data on how they evolved their extended lifespan and resisted cancer.

Previous studies found reduced GH-IGF1 signaling associated with increased resistance to cancer (Seim *et al.*, 2013). Additionally, it has been reported that long-lived bats have resilient telomeres that remain long despite advanced age (Foley *et al.*, 2018). Also, bats do not show an increased level of mitochondrial damage given their metabolic rate (Jebb *et al.*, 2018), suggesting that this group evolved adaptations in their DNA repair and maintenance mechanisms. These molecular adaptations were underpinned by a study showing that bats exhibit a unique age-related regulation of genes associated with DNA repair, immunity, and tumor suppression that underlies extended bat longevity (Huang *et al.*, 2019). Furthermore, this study reported that long-lived bats possess specific miRNAs that function as tumor suppressors. This provides a new potential molecular mechanism to decrease cancer risk not yet identified in any other lineage (Huang *et al.*, 2019). Finally, a recent study achieved a reference-level genome for six bat species. From an immune perspective, the high-quality dataset revealed exciting findings that could account for the pathogen-tolerant phenotype that distinguishes bats from other mammals (Jebb *et al.*, 2020). As an efficient immune system is tightly related to cancer resistance, this is also likely to be a pathway to be further investigated on cancer resistance in bats.

Carnivores

Carnivores exhibit extensive variation in body size, with some gigantic species, such as walruses and polar bears, also long-lived mammals (De Magalhães and Costa, 2009). However, the molecular mechanisms for maintaining large sizes and longevity in large carnivores have not been extensively investigated thus far. Huang *et al.* (2021) reported 100 body size-associated genes in carnivores related to cancer control, including tumor suppressors, DNA repair, and immunity. From these genes, 15 cancer-related genes were identified as rapidly evolving in the extremely large lineages, which might protect the animal from cancer invasion: ADGRF2, CABCOCO1, CATSPERG, CCDC146, CPLX4, CTLA4, MAS1, PACSIN1, PHF13, SDR39U1, SLC25A28, TCTE1, TERB1, YTHDC2 and ZBED1 (Huang *et al.*, 2021).

Other nonmammalian vertebrates

Information on the prevalence of cancer in wildlife vertebrates is still lacking, and this scarcity is even more pronounced in nonmammalian vertebrates. Nevertheless, it has been reported that tumors are more common in mammals than in other vertebrates, even though the information is limited (Effron *et al.*, 1977; Kitsoulis *et al.*, 2020).

Among reptiles, the genome of Lonesome George tortoise (*Chelonoidis abingdonii)* presents the contribution of multiple gene copy-number alterations in protein-coding genes with functions related to cancer resistance (SMAD4 and NF2) as well as giant-tortoise-specific duplications affecting two putative proto-oncogenes (MYCN and SET) (Quesada *et al.*, 2019). These findings may reflect a potential role in protecting against cancer in this long-lived and giant lineage of turtles. However, despite presenting several lineages with gigantism and great longevity, chelonians have not yet been the focus of studies to unravel the molecular mechanisms behind cancer resistance. Crocodiles and alligators are also good candidates to investigate cancer resistance, as cancer in these species was rarely reported. Moreover, some authors recently reported that a peptide derived from crocodiles’ leukocytes could kill human cancer cells via apoptosis induction (Maraming *et al.*, 2018).

Despite the high metabolic rate and the high cell turnover (Holmes and Ottinger, 2003), Møller *et al.* (2017) found a very low incidence of tumors in wild birds. This unexpected finding suggests that birds are more efficient in controlling the spread of tumor cells and that Peto's paradox may not apply to birds, as the authors identified a positive relationship between the incidence of tumors and body mass after controlling for critical life-history characteristics such as developmental rates and immunity (Møller *et al.*, 2017). Additionally, they found a negative relationship between the incidence of tumors and developmental rates, and the authors argue that this slow developmental rate would result in a more competent immune system, resulting, in turn, in a decrease in cancer incidence.

In amphibians, reports of cancer are rare. Interestingly, they appear to be naturally resistant to chemically induced malignancy (Taylor *et al.*, 2003). This could be due to a protective mechanism, given their ability to rapidly undergo apoptosis in response to antigens that may promote cancer (Taylor *et al.*, 2003). Another exciting study reported a natural peptide derived from the South American orange-legged leaf frog (*Pithecopus hypochondialis*) exhibiting antimicrobial and anticancer properties (Huang *et al.*, 2017). These two reports indicate that research on cancer-resistant amphibians should be more explored.

## Summary

Investigations focusing on Peto's paradox have extensively contributed to our knowledge of natural anticancer mechanisms. They unraveled evolution's general and specific solutions to the cancer problem in several lineages, especially those long-lived, large sizes, or both. Mechanisms such as positive selection of tumor suppressor genes, duplications, functional pseudogenization and alteration in the promoter regions of these genes, the action of transposable elements, early activation of crucial metabolic pathways for the arrest of cell division, changes in the epigenome, induction of apoptosis in cases of loss of tumor suppressor genes, replicative senescence, telomere resilience and the presence of tumor suppressor miRNAs were described as strategies for cancer resistance in different species.

Despite advances in research on natural cancer resistance, there is still a considerable gap in our knowledge of the phylogenetic diversity of tumor-suppressing mechanisms, as many reports have explicitly focused on a few mammalian species, such as elephant and naked mole rats. For example, Vincze *et al.* (2022) recently reported that cancer risk is lowest among ruminants, showing that members of the order Artiodactyla are likely good candidate models to study mechanisms of cancer resistance. However, to our knowledge, there is still no study focusing on these cancer-resistant animals. Furthermore, the last decade has shown several important discoveries on anticancer mechanisms found in other species. Considering the whole tree of Life that remains to be explored, many novel cancer defense mechanisms await to be discovered.

Besides the need to include many additional species from the tree of Life in cancer research, there is also a need for monitoring cancer in wildlife, especially in the context of current environmental perturbation, as this information will provide valuable insights into the impact of cancer as a severe threat to animal welfare and novel insights into new mechanisms of cancer resistance across species that may lead to the discovery of pressures that drive cancer transformation and about the nature of tumorigenesis itself. The potential to deepen our understanding of cancer biology also leads to the potential for new avenues in cancer therapy, as studies that link comparative evolutionary biology with molecular mechanisms of cancer resistance may guide new promissory treatments and prevention, even though it remains an important and open question whether the anticancer methods evolved in other mammals might benefit humans, both in general terms and specifically.

While the validity of Peto's Paradox is not universally accepted (see Ducasse *et al.*, 2015), it highlights the importance of applying evolutionary concepts to cancer studies, which can be instrumental for our understanding of cancer and its treatment. As the examples above show, natural selection has likely equipped species with the ability to suppress tumors to maintain cell function in evolutionary time. These adaptations are encoded in species genomes and hold great promise for new cancer suppression mechanisms. Incorporating evolutionary thinking and a phylogenetic approach into cancer research is necessary and especially possible with the increased availability of genomic and expression data in nonmodel organisms. To date, the data show how evolution can be creative in cancer prevention mechanisms, which makes comparative studies more interesting than ever and highlights the importance of a more intense integration of scientists working on oncology, ecology, and evolutionary biology.
